# Fecal microbiota transplantation in systemic sclerosis: A double-blind, placebo-controlled randomized pilot trial

**DOI:** 10.1371/journal.pone.0232739

**Published:** 2020-05-21

**Authors:** Håvard Fretheim, Brian K. Chung, Henriette Didriksen, Espen S. Bækkevold, Øyvind Midtvedt, Cathrine Brunborg, Kristian Holm, Jørgen Valeur, Anders Heiervang Tennøe, Torhild Garen, Tore Midtvedt, Marius Trøseid, Hasse Zarè, May Brit Lund, Johannes R. Hov, Knut E. A. Lundin, Øyvind Molberg, Anna-Maria Hoffmann-Vold

**Affiliations:** 1 Department of Rheumatology, Oslo University Hospital, Rikshospitalet, Oslo, Norway; 2 Institute of Clinical Medicine, University of Oslo, Rikshospitalet, Oslo, Norway; 3 Norwegian PSC Research Center, Department of Transplantation Medicine, Oslo University Hospital, Oslo, Norway; 4 Research Institute of Internal Medicine, Oslo University Hospital, Rikshospitalet, Oslo, Norway; 5 Department of Pathology and Centre for Immune Regulation, Oslo University Hospital, Oslo, Norway; 6 Oslo Centre for Biostatistics and Epidemiology, Research Support Services, Oslo University Hospital, Oslo, Norway; 7 Unger-Vetlesen Institute, Lovisenberg Diaconal Hospital, Oslo, Norway; 8 Department of Gastroenterology, Oslo University Hospital, Oslo, Norway; 9 Department of Microbiology, Tumor and Cell Biology, Karolinska Institutet, Stockholm, Sweden; 10 Section of Clinical Immunology and Infectious Diseases, Oslo University Hospital, Rikshospitalet, Oslo, Norway; 11 Clinical Trial Unit, Oslo University Hospital, Rikshospitalet, Oslo, Norway; 12 Department of Respiratory Medicine, Oslo University Hospital, Oslo, Norway; 13 K. G. Jebsen Centre for Coeliac Disease Research, University of Oslo, Oslo, Norway; VU University Medical Center, NETHERLANDS

## Abstract

**Objectives:**

Systemic sclerosis (SSc) is an auto-immune, multi organ disease marked by severe gastrointestinal (GI) involvement and gut dysbiosis. Here, we aimed to determine the safety and efficacy of fecal microbiota transplantation (FMT) using commercially-available anaerobic cultivated human intestinal microbiota (ACHIM) in SSc.

**Methods:**

Ten patients with SSc were randomized to ACHIM (n = 5) or placebo (n = 5) in a double-blind, placebo-controlled 16-week pilot. All patients had mild to severe upper and lower GI symptoms including diarrhea, distention/bloating and/or fecal incontinence at baseline. Gastroduodenoscopy transfer of ACHIM or placebo was performed at weeks 0 and 2. Primary endpoints were safety and clinical efficacy on GI symptoms assessed at weeks 4 and 16. Secondary endpoints included changes in relative abundance of total, immunoglobulin (Ig) A- and IgM-coated fecal bacteria measured by 16s rRNA sequencing.

**Results:**

ACHIM side effects were mild and transient. Two placebo controls experienced procedure-related serious adverse events; one developed laryngospasms at week 0 gastroduodenoscopy necessitating study exclusion whilst one encountered duodenal perforation during gastroduodenoscopy at the last study visit (week 16). Decreased bloating, diarrhea and/or fecal incontinence was observed in four of five patients in the FMT group (week 4 or/and 16) and in two of four in the placebo group (week 4 or 16). Relative abundance, richness and diversity of total and IgA-coated and IgM-coated bacteria fluctuated more after FMT, than after placebo.

**Conclusions:**

FMT of commercially-available ACHIM is associated with gastroduodenoscopy complications but reduces lower GI symptoms by possibly altering the gut microbiota in patients with SSc.

## Introduction

Systemic sclerosis (SSc) is a complex, multi-organ disorder characterized by immune-mediated inflammation, progressive organ fibrosis and vascular pathology [1]. Severity and extent of GI involvement varies within the SSc population, but overall, more than 90% of patients report GI symptoms [2]. The most commonly reported findings are reduced esophagus motility, gastroesophageal reflux disease (GERD), reduced intestinal motility, small intestine malabsorption and fecal incontinence [3, 4]. The mechanisms behind the GI affection in SSc are not well understood, but appear multifactorial [5, 6]. Previous studies show that intestinal microbiota composition in SSc differs from healthy individuals [7, 8].

To date, effective treatment alternatives for SSc-related GI disease are lacking and mostly limited to providing partial symptom relief [9, 10]. Fecal microbiota transplantation (FMT) is getting increasing attention as a potential therapeutic intervention for several diseases showing a good safety profile and relevant clinical effects; but it has not been assessed in rheumatic diseases, including SSc [11, 12]. One of the main challenges in prior FMT studies was donor-dependent variation of the fecal bacteria which could be overcome by using a standardized bacterial culture across all FMTs [13–15].

Herein, we performed a first-in-man fecal microbiota transplantation (FMT) pilot study with commercially-available anaerobic cultivated human intestinal microbiota (ACHIM) in patients with SSc to determine safety, effects on GI symptoms and on fecal microbiota composition.

## Materials and methods

### Study design and participants

This was a single center randomized double-blind placebo controlled pilot trial with active intervention by a standardized FMT culture over 16 weeks with six study visits conducted at Oslo University Hospital between January and May 2018 (See S1 Fig). Patients were eligible for the study if they were between 18 and 70 years old, fulfilled the 2013 American College of Rheumatology/European League against Rheumatisms SSc classification criteria [16], and had clinically apparent upper and lower GI involvement (defined below). Study participants were recruited from the Oslo University Hospital rheumatology outpatient clinic from August to December 2017. To decrease the heterogeneity of the study participants we chose patients of female gender and with limited cutaneous SSc [17]. For list of exclusion criteria`s, see S1 File.

### Registration

The trial protocol was approved by the Regional Committees for Medical and Health Research Ethics (REK) on September 8, 2016 (Approval No: 2016/1529) and followed the Helsinki Declaration. All patients gave after verbal information written consent before study start. The study was registered at clinicaltrials.gov (NCT03444220), one month after study start while still all participants and staff was blinded. The authors confirm that all ongoing and related trials for this drug/intervention are registered.

### Randomization and masking

Eligible participants were randomly assigned to treatment. An unblinded staff member (TG) who was not otherwise involved in the clinical aspects of the study generated the random sequence for treatment assignment. All patients and care providers responsible for assessing patients during the study and outcomes were blinded. Active treatment and placebo were packaged, and handled so that participants and study staff were not able to distinguish between treatments. Participants, study staff performing safety and efficacy assessments and analyzing safety and other trial data were blinded to group assignment until the database was locked.

### Procedures and treatment

Treatment was installed during gastroduodenoscopy twice with two weeks apart (week 0 and 2). In addition, the patients underwent a third gastroduodenoscopy at the last study visit (week 16) for sampling of biological material (biopsies). The gastroscopy procedure is explained in further detail in S1 File. The active treatment arm received the patented single-donor ACHIM developed by ACHIM Biotherapeutics AB, Sweden (S5 File); the placebo group received ACHIM bacteria medium, stored and thawed following the same procedure as ACHIM but without fecal microbiota. The installation procedure was identical in both treatment arms. We use the familiar acronym FMT when referring to fecal microbiota transplantation with ACHIM throughout the manuscript.

### Safety, feasibility, efficacy and clinical assessment

Safety was assessed at each study visit and collected by a standardized safety form and any unfavorable and unintended sign, symptom, or disease temporally associated with the study treatment was registered as an adverse event (AE). A serious AE (SAE) was defined as a state that required hospitalization, death or life threatening situations, or disability or reducing the patient’s capacities.

Efficacy was evaluated by patient-reported outcome using the ULCA GIT 2.0 score questionnaire (UCLA GIT score) and was done at each study visit [18]. The UCLA GIT score is a seven-item scale measuring upper and lower GI symptoms including reflux, distention/bloating, diarrhea, fecal incontinence, constipation; and emotional well-being, and social functioning. The UCLA GIT score is explained in further detail in S1 File. Additionally, the overall presence of disabling fecal incontinence, defined as being unable to control bowel movement leading to fecal soilage, prior to study inclusion was noted by an SSc expert (AMHV) and registered at every study visit. For information on secondary clinical efficacy assessments, see S1 File.

Collection procedures of biological material and details on analysis of short-chain fatty acids (SCFAs), relative abundance of fecal bacteria and Ig-coating is described in S1 File.

### Outcomes

The primary endpoint was safety and clinical efficacy. Safety was collected by a standardized safety form. The primary efficacy endpoint was met if the patients showed improvement of clinical apparent GI involvement at baseline after FMT; measured with the previously validated minimally clinically important difference (MCID) values of the UCLA GIT score (below) [19]. Secondary and explorative endpoints included effect on overall presence of disabling fecal incontinence, changes in the gut microbiome composition, relative abundance of IgA and IgM coated fecal bacteria (to probe interactions between the gut microbiota and the immune system), fecal calprotectin levels and fecal SCFA concentrations; changes in the modified Rodnan skin score, lung function, CRP, ESR, and patient and physician global. Items were assessed as changes from week 0 to week 4 and/or week 0 to week 16.

### Statistical analysis, sequence processing and bioinformatics

All analyzes were performed using IBM SPSS Statistics version 25 and STATA version 15. Due to the small sample size, solely descriptive statistics without p-values were applied to clinical data analysis.

The total UCLA GIT score, and the seven single items were assessed at week 0 and the MCID from week 0 to week 4 and week 16. Changes from mild or moderate-severe GI symptoms at week 0 were defined as clinical meaningful if the MCID, the smallest change in score that patients perceive as beneficial, met the “somewhat better” threshold, as previously published [20]. Linear mixed models analysis were used to estimate changes in the microbiota composition and the changes in IgA and IgM coating patterns over the entire follow-up period (i.e. baseline, 4 weeks, and 16 weeks) and to control for repeated measurements (details in S1 File).

## Results

### Clinical and demographic characteristics of pilot study participants

**Fig 1:**
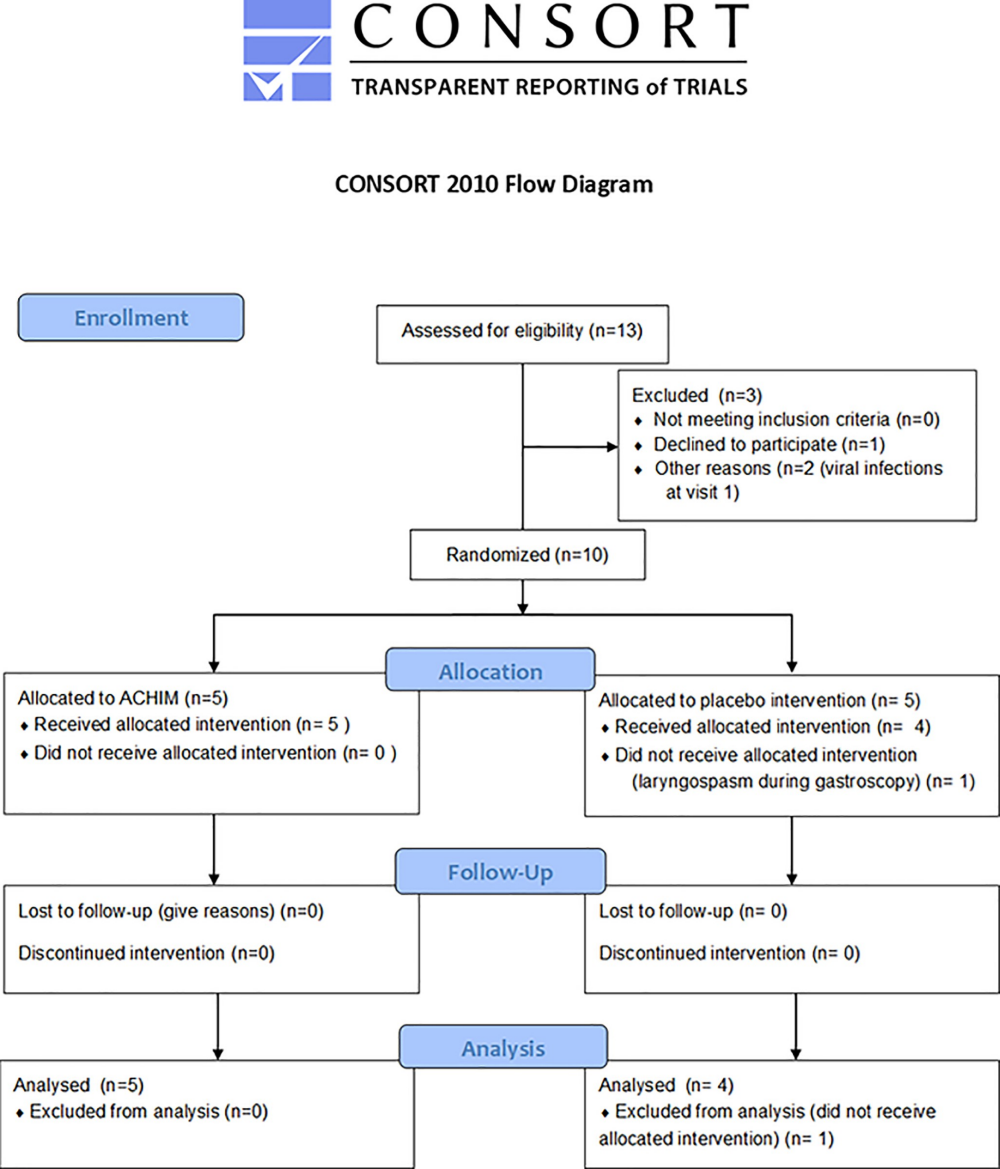
CONSORT flow chart.

**Fig 2:**
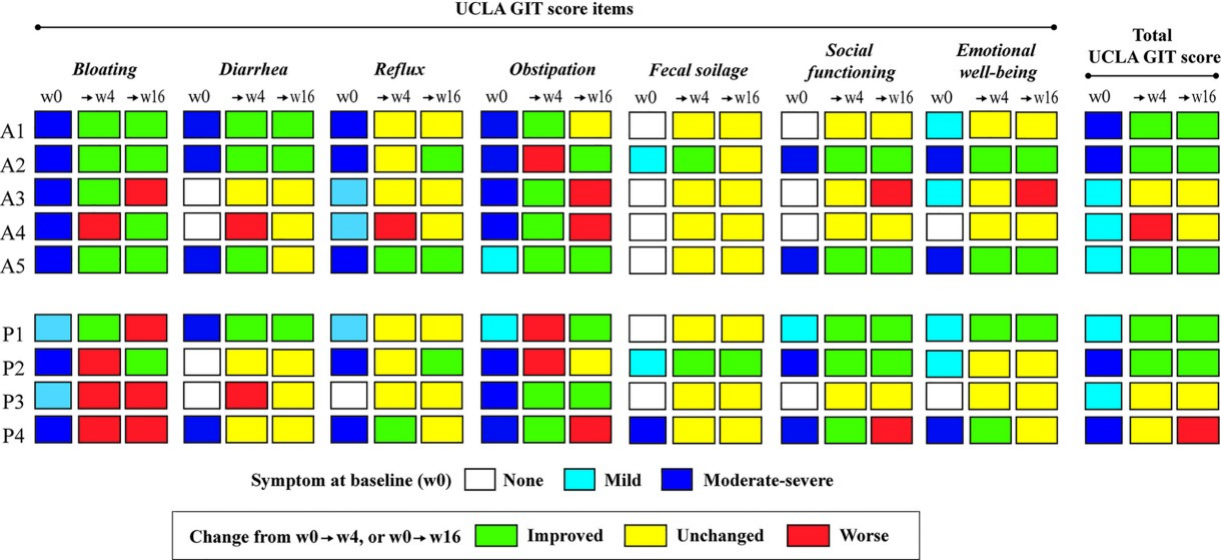
Clinical efficacy of fecal microbiota transplantation (FMT) on gastrointestinal (GI) symptoms. Efficacy of FMT measured by the total UCLA GIT score and all seven items of the UCLA GIT score (bloating, diarrhea, reflux, obstipation, fecal soilage, social functioning and emotional well being). Patients receiving FMT (active treatment) marked as A and placebo marked as P. Symptoms at baseline (week 0; w0) are segregated into dark blue (moderate-severe), light blue (mild) and white (no) GI symptoms. Changes to week 4 and week 16 are marked in green (improved), yellow (stable) or red (worsening).

Thirteen eligible SSc patients were screened and 10 were enrolled in the study; with five patients assigned to active intervention and five to placebo (Fig 1). Out of the three patients not enrolled, two patients were excluded from the study due to ongoing viral infections at visit 1 and one patient withdrew from the study due to personal reasons. One of the patients assigned to placebo was per protocol excluded from the study after the first gastroduodenoscopy due to laryngospasms. The remaining nine SSc patients completed all study protocol procedures and were included in the analyses of outcome measures. Disease features and treatment of these nine patients are shown in Table 1, while patient reported GI symptoms at baseline are described in Fig 2.

**Table 1:** Demographics, disease features and ongoing therapies of the nine systemic sclerosis patients who completed the ReSScue pilot study.

Characteristics at baseline	Total study cohort (n = 9)	FMT intervention (n = 5)	Placebo intervention (n = 4)
Age, years	62 (5.7)	58 (5.6)	66 (1.5)
Female gender	9 (100)	5 (100)	4 (100)
Body Mass Index	24.0 (2.3)	23.6 (2.5)	24.4 (2.1)
Disease duration, years	12.0 (10.6)	7.4 (6.7)	17.8 (12.6)
Limited cutaneous SSc	9 (100)	5 (100)	4 (100)
Anti-centromere antibodies	8 (89)	5 (100)	3 (75)
mRSS, median (IQR)	4 (2–5)	4 (2–8)	3 (2–4)
DU	0 (0)	0 (0)	0 (0)
FVC, %	97 (11)	98 (12)	97 (12)
DLCO, %	74 (14)	76.4 (12)	72 (18)
Hemoglobin, g/dL	13.1 (0.8)	13.1 (0.8)	13.1 (1.0)
CRP, mg/L	5 (6.7)	7 (8.8)	4 (3.1)
ESR, mm	13 (6.9)	14(5.9)	13 (9.0)
UCLA GIT score	0.72 (0.5)	0.68 (0.4)	0.77 (0.6)
Patient global assessment, cm	3.9 (2.7)	3.0 (2.8)	4.8 (2.6)
Physician global assessment, cm	2.2 (2.1)	2.4 (2.8)	2.1 (1.0)
**Ongoing Treatment**			
Immune modulating drugs	2 (22)	1 (20)	1 (25)
Proton pump inhibitors	7 (78)	3 (60)	4 (100)
Calcium channel blockers	7 (78)	4 (80)	3 (75)
Endothelin receptor antagonist	2 (22)	2 (40)	0 (0)

Values are n (%) for categorical variables and mean (SD) for continuous variables. FMT: in vitro cultured fecal microbiota transplantation; IQR: interquartile range; n: number; SSc: systemic sclerosis; mRSS: modified Rodnan Skin Score; DU: digital ulcer; FVC: Forced Vital Capacity; DLCO: diffusion capacity of the lung carbon monoxide; CRP: C-reactive protein; ESR: erythrocyte sedimentation rate, UCLA GIT score: University of California Los Angeles Gastrointestinal score; Immune modulating drugs used by the study particitpants included prednisone, methotrexate and rituximab (all medications use was registered)

### Safety profile of FMT in SSc

No patients experienced fever or signs of infection after the interventions. Patients in the FMT group (n = 5) reported more AEs post-intervention than the placebo controls (n = 4), but all the AEs were regarded as mild and transient, including abdominal bloating, diarrhea, nausea and constipation (Table 2).

**Table 2:** Cumulative incidence of adverse events by week 4 and week 16 in the ReSScue pilot study.

	By week 4		At week 16	
	FMT	Placebo	FMT	Placebo
Total adverse events, n (%)	5 (100)	3 (75)	5 (100)	4 (100)
Severe adverse events, n (%)	0 (0)	0 (0)	0 (0)	1 (25)
Mild adverse events, n (%)	5 (100)	3 (75)	5 (100)	3 (75)
Bloating	2 (40)	2 (50)	5 (100)	1 (25)
Diarrhea	2 (40)	1 (25)	3 (60)	2 (50)
Constipation	1 (20)	0 (0)	5 (100)	1 (25)
Nausea	3 (60)	1 (25)	4 (80)	2 (50)
Vomiting	0 (0)	1 (10)	1 (0)	1 (0)
Abdominal discomfort	4 (80)	0 (0)	5 (100)	0 (0)
Fever	0 (0)	0 (0)	0 (0)	1 (25)

The patient who experienced laryngospasm during the first gastroduodenoscopy, necessitating exclusion from the rest of the study, is excluded from the table. FMT: in vitro cultured fecal microbiota transplantation, n: number

There were two procedure-related serious adverse events in two patients, both of which had been assigned to placebo. The first SAE was laryngospasms during the baseline gastroduodenoscopy, which necessitated per protocol exclusion from the study (as indicated above). The patient recovered without any sequela. The second SAE was duodenal perforation that occurred during the final gastroduodenoscopy, at the last study visit (week 16) after completing all other study visits. This resulted in hospitalization and treatment with intravenous antibiotics. The patient recovered without any form of sequela.

One of the patients assigned to the placebo group (P2; Fig 2) had moderately elevated liver enzymes at screening and baseline. We did not exclude her from the study, but referred her to the Department of Gastroenterology for diagnostic work up. She was diagnosed with primary biliary cholangitis (PBC) at week 3 in the study period. Ursodeoxcholic acid (250mg x3) was started from week 10. This patient reported improved GI symptoms, particularly at week 16 (P2; Fig 2).

### Effects of FMT on clinical outcome measures

At the time of the primary intervention (week 0), all the nine study completers had clinical apparent GI symptoms, consistent with actual presence of SSc-related upper and/or lower GI symptoms (shown as light blue (mild) or dark blue (moderate to severe) in Fig 2). At week 4, three patients in the FMT group and one in the placebo group reported an improvement in overall (upper and lower) GI symptoms that was consistent with clinically meaningful reduction in the total UCLA GIT score (shown in green in Fig 2). These improvements continued up to week 16 in all three patients (Fig 2). One patient in the FMT group had worsening (shown in red in Fig 2) of total GIT score on week 4 but the score returned back to its baseline value at week 16 (shown in yellow in Fig 2).

**Fig 3:**
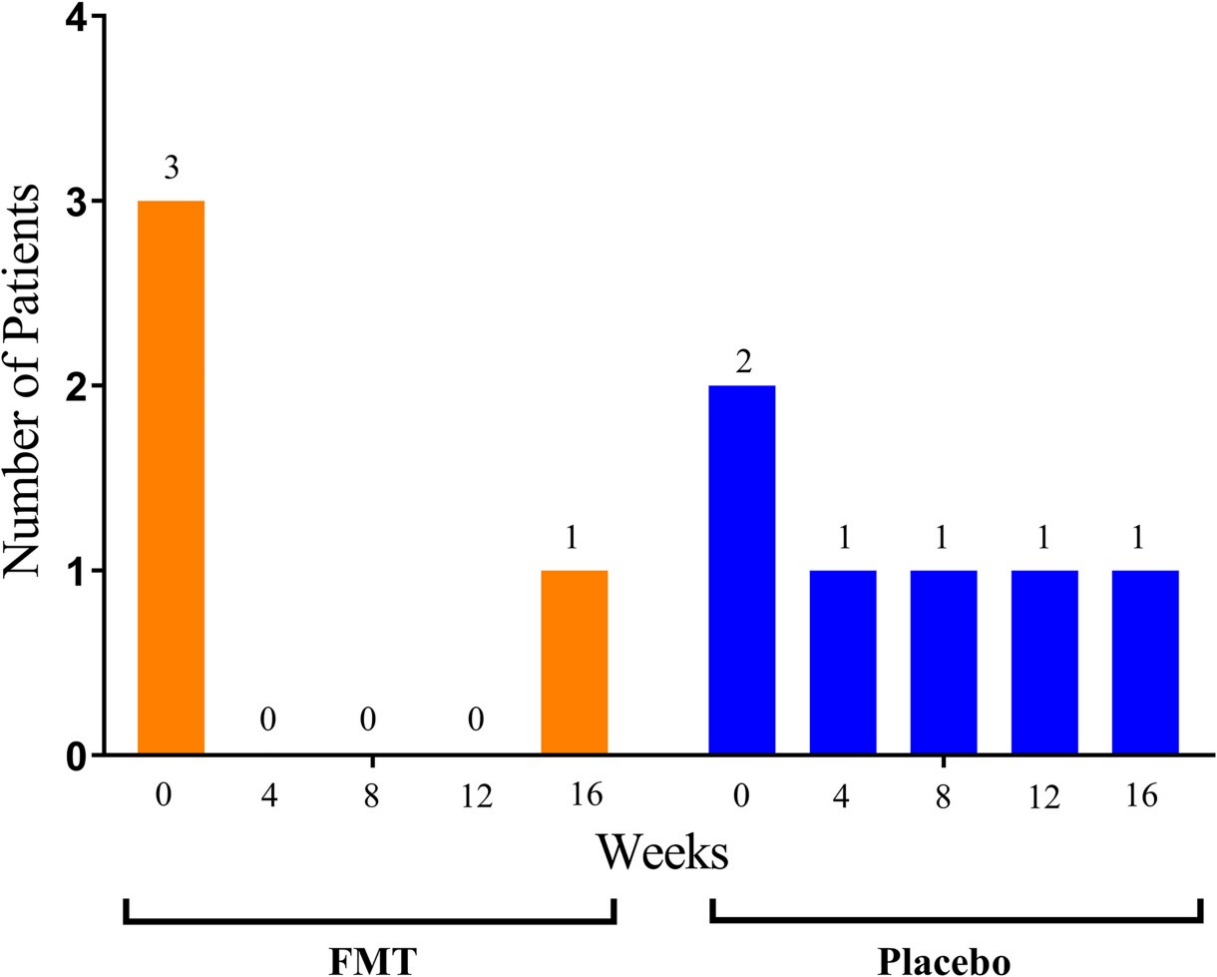
Changes in patient reported fecal incontinence. Patient reported fecal incontinence was registered at week 0, 4, 8, 12 and 16 as an exploratory endpoint. Three patients in the FMT group (orange) had fecal incontinence at week 0, with restoration of incontinence at week 4. Two patients in the placebo group (blue) had fecal incontinence at week 0, with restoration of incontinence within week 4 in one patient.

Effects of the intervention appeared to be most pronounced in patients presenting with lower GI symptoms at baseline such as bloating, diarrhea, and/or fecal incontinence. Specifically, at week 4, four of five patients in the FMT group reported improvement in bloating (Fig 2). At week 16, bloating was improved in four of five FMT patients and only one of four placebo controls. Diarrhea improved at week 4 in three out of the three with diarrhea at baseline receiving FMT and one of two in the placebo group (Fig 2). At week 0, five patients (three in the FMT group and two placebo controls) reported severe and disabling fecal incontinence which was not fully captured by the UCLA GIT score. At week 4, major improvement in fecal incontinence was reported by all three patients in the FMT group, compared to one of two placebo controls (Fig 3). Additionally, we recorded a number of secondary clinical outcome measures, summarized in Table 3.

**Table 3:** Secondary clinical outcome measures in the ReSScue pilot study.

Explorative outcome measure	FMT (n = 5)	Placebo (n = 4)
mRSS	-0.8 (-2.4 to 0.8)	-0.8 (-2.3 to 0.8)
DU, new onset	0 (0)	0 (0)
FVC %	-0.4 (-9.2 to 8.4)	-1.5 (-11.7 to 8.7)
DLCO %	-2.0 (-6.8 to 2.8)	-6.3 (-11.8 to -0.7)
CRP, mg/l	-4.6 (-15.0 to 5.8)	-1.5 (-7.4 to 4.4)
ESR, mm/h	1.4 (-11.6 to 14.4)	-4.0 (-15.0 to 7.0)
Patient global assessment, cm	0.0 (-6.4 to 6.4)	-2.5 (-7.8 to 2.8)
Physician global assessment, cm	-0.6 (-3.2 to 2.0)	-0.35 (-3.7 to 3.0)

Results are shown as relative change in the individual measures from week 0 to week 16 with 95% CI. Values are n (%) for categorical variables and mean (95%CI) for continuous variables. CI: confidence interval; FMT: in vitro cultured fecal microbiota transplantation; mRSS: modified Rodnan Skin Score; DU: digital ulcers; FVC: forced vital capacity; DLCO: diffusing lung capacity for carbon monoxide; CRP: C-reactive protein; ESR: Erythrocyte sedimentation rate

### Effects of FMT on fecal calprotectin and SCFA levels

We observed relatively stable fecal calprotectin levels in the placebo group at week 0, 4 and 16, but an increase in calprotectin levels in all five FMT treated patients either at week 4 or 16 (S2 Fig). We did not identify any distinct pattern of changes in any of the measured SCFAs after FMT at week 4 or 16 (S4 File).

### Effects of FMT on the fecal bacteria composition and IgA and IgM coated fractions

**Fig 4:**
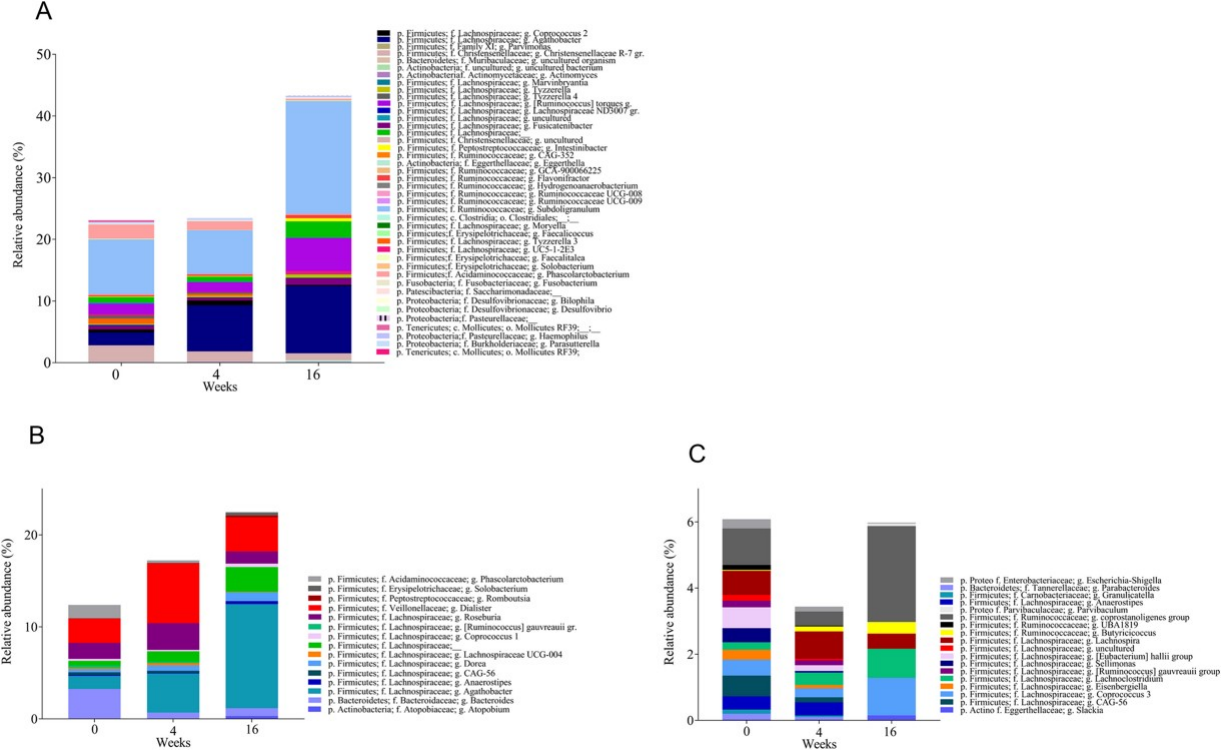
Relative abundance of total, IgA and IgM coated fecal bacteria in the FMT group. Genera that showed increased relative abundance after FMT were predominantly within the Firmicutes phylum, including genera within the Ruminococcaceae and Lachnospiraceae families. At baseline, relative abundance of IgA and IgM coated fecal bacteria were similar in the FMT and placebo groups. IgA and IgM coating pattern at genera level changed from baseline to weeks 4 and 16 in the FMT group, while the placebo group showed stable relative abundances of these genera. A: Relative abundance of unsorted bacteria. Only bacteria with change (p<0.1) in relative abundance of unsorted bacteria from week 0–4 or week 0–16 are shown. B: Relative abundance of IgA coated bacteria. Only bacteria with changes (p<0.1) in relative abundance of IgA coating from week 0–4 or week 0–16 are shown. C: Relative abundance of IgM coated bacteria. Only bacteria with change (p<0.1) in relative abundance of IgM coating from week 0–4 or week 0–16 are shown.

At baseline, there were no significant differences in fecal bacteria composition between the FMT and placebo groups, as measured by intra-individual (alpha) diversity (Shannon index, phylogenetic diversity, number of distinct operational taxonomic units (OTUs)) and global microbiota composition (beta diversity measured by unweighted unifrac analysis)(See S3 Fig). At week 16, but not at week 4, we identified differences in fecal bacteria composition between the FMT and placebo groups by the beta diversity (p<0.02) and number of OTUs (p<0.006). At the phylum level, there were no clearly altered relative abundances observed after FMT. We did find changes in relative abundances of several genera at weeks 4 and 16 in the FMT group (Fig 4) while the placebo group showed stable relative abundances of these genera. Genera that showed increased relative abundance after FMT were predominantly within the Firmicutes phylum, including genera within the Ruminococcaceae and Lachnospiraceae families. We also identified changes of relative abundancies within IgA and IgM coated bacteria at genera from baseline to weeks 4 and 16 in the FMT group, while the placebo group showed stable relative abundances of these genera; i.e. Parabacteroides, Anaerostipes, Escherichia-Shigella, Coprococcus, Roseburia, Dialister and Anaerostipes (Fig 4).

## Discussion

SSc is marked by high cumulative incidence of severe and hitherto untreatable GI involvement, and alterations of the fecal microbiota composition [2, 8]. In this study, we show that FMT by an in vitro cultured standardized single donor bacterial mixture is safe and reduces lower GI symptoms in patients with SSc. To our knowledge, this is the first in man randomized clinical trial assessing both safety and efficacy of FMT in patients with a systemic rheumatic inflammatory disease.

The observed side effects directly related to the FMT by ACHIM were only short lasting and minor, and similar to previously reported FMT side effects [21]. It is important to note that we experienced two severe adverse events related to the enteric administration by endoscopy. This may be due to increased vulnerability of SSc patients marked by a fibrotic and stiffer GI tract. Studies on FMT with capsulated microbiota given per oral are emerging in C. difficile colitis [22]; and this administration route appears attractive in SSc, given that it proves feasible to manufacture capsules for efficient delivery of the desired bacterial ecosystem.

In this study, the SSc patients mainly reported improvement in lower GI-symptoms. Especially several patients with long standing fecal incontinence had marked improvement of symptoms following FMT. Changes in upper GI-symptoms were less pronounced. Whether this is due to the small sample size, or has other reasons is unclear, but should be addressed in larger trials. As outcome measures we chose the UCLA GIT score because it (i) is the only validated GIT scoring system in SSc and (ii) had readily available definitions of meaningful clinically important difference, expressed as relative changes in the total and individual item scores. We did, however, experience limitations with the UCLA GIT score, particularly regarding identification and longitudinal assessment of fecal incontinence. The UCLA GIT questionnaire only captures symptoms present in the past week. This probably works fine for most items, but from our data set where fecal incontinence was registered as an ever present GI symptom, it seems that the UCLA GIT score underestimated the negative impact of this symptom at baseline, and failed to detect post-intervention changes that were described as important by the patients. Hence, in future studies there seem to be a need for fecal incontinence scores specifically designed to capture clinically meaningful changes. Notably, even though the AEs related to infusion of ACHIM or placebo were short lasting and transient, they were recorded as ever present since last study visit. The UCLA GIT score only covers GI symptoms the previous week. For this reason, there were occasional mismatches between recorded AEs and the UCLA GIT score.

The ideal study duration of FMT trials are unknown. We observed in some patients in this pilot trial that the FMT effects tended to wane, with recurrence of lower GI symptoms towards the end of the study period. The study period of 16 weeks was based on previous FMT trials in inflammatory bowel disease patients. But based on the results of this pilot trial one could argue that endpoints should be estimated at around 4 weeks for short term an at 12 weeks after FMT before recurrence of GI symptoms. The waning effect possibly reflects that gut microbiota in general appears to be resilient to change, and often returns to its pre-intervention state within weeks [23, 24]. Consequently, repeated interventions may be necessary to maintain a desired microbiota composition for clinical purposes. Also, the optimal donor composition for this and all other patient groups is unknown and may differ between patients. In addition, there is a lack of knowledge about the optimal dosage and interval of treatment [25]. Closing this knowledge gap could potentially further increase the effect of FMT leading to broad use of FMT in many auto-immune diseases in the future.

A major advantage of using ACHIM is that it ensures administration of the same bacteria to all study participants, and makes it possible to systemically track the donor-derived microbiome across all the FMT recipients. Given that FMT using ACHIM significantly reduced lower GI symptoms, we sought to determine if specific bugs found in ACHIM were enriched in feces following treatment. We observed post-intervention increase in the relative abundance of three bacterial families (Ruminococcaceae, Lachnospiraceae and Eggerthellaceae) which are dominant in ACHIM (personal communication Tore Midtvedt 2019). Although many of the genera present in ACHIM are known butyrate producers, we did not observe any significant post-intervention changes in SCFA, but this may be due to the small sample size.

Previous studies in murine models suggest activation of adaptive immune responses contributes to the efficacy of FMT [26]. To assess if FMT triggered adaptive immunity in patients with SSc, we sorted and sequenced IgA and IgM coated bugs from fecal samples and compared relative coating amongst FMT and placebo. We observed a change in the relative abundance of IgA and IgM coated bacteria after FMT. Some of the bacteria that were found to change their IgM coating pattern after FMT, like the genera Parabacteroides, Anaerostipes and Escherichia-Shigella, were present in the in vitro cultured fecal microbiota. Some of the bacteria which have previously been associated with other auto-immune diseases or pro-inflammatory status (Coprococcus, Roseburia, Dialister and Anaerostipes) were altered in their IgA coating patterns after FMT. These observations support the notion of dynamic and ongoing immune responses to gut bacteria, and indicate that acquisition of a different gut microbiota induce immunological changes, as reflected by alterations in the pattern and/or extent of IgA and IgM coating. Accordingly, we observed changes in calprotectin levels in the FMT group (but not in the placebo controls), possibly reflecting neutrophil mobilization in response to FMT. No patient performed colonoscopy in the screening phase of the study, and fecal samples for calprotectin measurement was stored for analysis after study completion. In hindsight, one should consider measuring calprotectin at screening of patients and perform colonoscopy if indicated.

In this study we did not focus on the mechanistic pathways by which FMT exerts its effects, but such future studies are highly warranted. One could speculate that there is a mechanistic link between dysmotility and dysbiosis in SSc; and that manipulation of gut microbiota with FMT could affect motility patterns, which in turn leads to improvement of GI symptoms.

This study has several limitations including the small number of participants and short study period. Some of the patients included had relatively mild GI symptoms, decreasing the chance of observing a clinical treatment effect. In addition, we only included female patients with longstanding limited cutaneous systemic sclerosis. This may also have led to the rather small changes that were observed in the secondary clinical outcome measures. A larger study is needed to further elucidate the findings from this promising proof-of concept study.

In conclusion, this first in line study indicates that FMT with in vitro standardized cultivated fecal microbiota is safe regarding the manipulation of the gut microbiota, but there are concerns regarding the safety of gastroduodenal administration. We observed alterations of the gut microbiota composition and Ig coating patterns in patients with SSc after FMT. The sample size was too small to make any meaningful conclusion regarding clinical effects of FMT on GI symptoms, but we observed clearly improvement in diarrhea, bloating and fecal incontinence.

## Supporting information

S1 File Supplementary material and methods. Additional information about material and methods.
(PDF)

S2 File CONSORT 2010 checklist. (DOC)

S3 File Study protocol. (DOCX)

S4 File Short chain fatty acids. Short chain fatty acids analysis at week 0, 4 and 16.
(XLSX)

S5 File Supplementary information about ACHIM. (PDF)

S6 File De-identified data sets used in the performed analysis. (ZIP)

S1 Fig Study design of fecal microbiota transplantation in systemic sclerosis. Six study visits over a period of 16 weeks. Intervention with ACHIM with two weeks apart (week 0 and 2). Pulmonary function tests performed at week 0 and 12. Fecal samples each week, and collected at all study visits. Clinical exam and UCLA GIT score performed at all study visits (week 0, 2, 4, 8, 12 and 16).
(PDF)

S2 Fig Calprotectin levels. Individual level of fecal calprotectin (mg/kg) at week 0, week 4 and week 16. A = active treatment group patient. P = placebo group patient.
(PDF)

S3 Fig Beta diversity. Individual beta diversity at week 0 (V1), week 4 (V3) and week 16 (V6).
(PDF)
