# Supplementary material for: Fecal microbiota transplantation in systemic sclerosis: A double-blind, placebo-controlled randomized pilot trial
Source: PLoS One. 2020 May 21;15(5):e0232739. doi: 10.1371/journal.pone.0232739 (PMC7241803; doi:10.1371/journal.pone.0232739)
Supplement: S1 File — Additional information about material and methods. (PDF) [file pone.0232739.s001.pdf]

Supporting information

Methods

Study participants, in- and exclusion criteria

Study participants were recruited from the Oslo University Hospital rheumatology outpatient clinic from August to December 2017. Disease duration was defined as the time from onset of first non-Raynaud symptom attributed to SSc until the first study visit. Patients could be on concomitant immunosuppressive medications if provided doses were stable for at least 3 months prior to inclusion and were not likely to change during the study. Major exclusion criteria were (i) systemic antibiotic treatment within last 6 weeks, (ii) severe end organ disease (lung disease with forced vital capacity (FVC) <50% and/or diffusing lung capacity for carbon monoxide (DLCO) <40%, severe heart failure with ejection fraction<30% or end stage kidney disease with glomeration filtration rate <30 ml/min) and (iii) active digital ulcers, due to increased risk of antibiotics use.

Gastroduodenoscopy procedures

Patients were examined by a trained endoscopist applying standard technique using Olympus endoscopes. Eight biopsies were sampled from the descending part of the duodenum using biopsy forceps. ACHIM was administered by a through-the-scope catheter distal for the biopsy site, followed by 30mL of saline.

Efficacy and clinical assessment

The UCLA GIT score was applied for efficacy assessment as the primary outcome. All scales are scored from 0 (better) to 3 (worse) except the diarrhea and constipation scales (ranges are 0–2.0 and 0– 2.5). The total UCLA GIT score is the sum of all scales (except constipation) converted into a score ranging from 0.00–2.83 providing an estimation of the

severity of GI involvement. The total UCLA GIT score and each individual domain has previously been validated. Clinical GI involvement was defined as apparent if the patients reported symptoms resulting in a total score or at least one of the five GI items ≥0.01 and was segregated into mild (<0.5 or for fecal incontinence and distention/bloating <1.01) or moderate-severe GI symptoms (≥0.5 or for fecal incontinence and distention/bloating ≥1.01) [1].

Secondary clinical efficacy assessments were determined at each study visit including patient and physician global assessments (Supplementary material Figure 1). Clinical examinations were performed, and blood samples (including erythrocyte sedimentation rate (ESR) and C-reactive protein (CRP)), drawn after standard procedures at each visit. Modified Rodnan Skin Score and digital ulcers were assessed at all visits by the same investigators (AMHV, HF). Pulmonary function tests were carried out according to the American Thoracic Society/European Respiratory Society guidelines, using Jaeger Master Screen Body (Eric Jaeger, Wurzburg, Germany) at week 0 and 12 [2]. Use of antibiotics and medications, change in medication and change in nutrition was noted at all visits.

Biological material collection and analysis

The study participants collected weekly fecal samples at home, using an easy-to-use collecting device (Protocult™) [3], stored the samples immediately at -20 °C in sterile containers without additives and brought their frozen fecal samples to the study site at each study visit. At the study site, the samples were stored immediately at –80°C. Fecal calprotectin was measured in stool samples from week 0, 4 and 16 using a standard ELISA assay. Fecal concentrations of short-chain fatty acids (SCFAs), including major SCFAs (acetic, propionic, and butyric acids), and minor SCFAs (iso-butyric, valeric, iso-valeric, caproic and iso-caproic acids), were analyzed with gas chromatography and quantified using

flame ionization detection, as previously described [4]. A modified version of BUGFACS (below) was applied to assess relative abundance of bacterial genera in IgA and IgM coated fractions as well as total (unsorted) fecal bacteria [5]. Library preparations were performed in accordance with an established protocol with amplification of the V3-V4 region of the 16S rRNA gene and sequencing on the Illumina MiSeq platform [6].

IgA and IgM binding patterns protocol

We applied a modified method adopted from Planer et al. [7]. Briefly, frozen fecal samples were suspended in PBS. 5 mg of feces passes through a mesh filter, ice cold PBS was added and centrifuged at 10,000 *g*. The cell pellet re-suspended in FACS buffer and aliquoted into 5 tubes before addition of anti-IgM PE, anti-IgG PE, anti-IgA PE, anti-IgA isotype control or PBS. SytoBC bacterial DNA stain was added and before sort using FACS. For each sample, 50,000 ‘events’ were recovered. Each fraction was stored at −20°C before 16S rRNA sequencing.

Statistical analysis, sequence processing and bioinformatics

Linear mixed models analysis were used to estimate changes in the microbiota composition and the changes in IgA and IgM coating patterns over the entire follow-up period (i.e. baseline, 4 weeks, and 16 weeks) and to control for repeated measurements. Time, group (FMT or placebo), and time-by-group interaction were fixed effects in all models. All models included random intercept. Based on the linear mixed models, we estimated mean treatment group values with 95% CIs for the three time points (baseline, 4 weeks, and 16 weeks) for each group. We also estimated the mean group changes from baseline to 4 and 16 weeks and the between-group difference in change from baseline to 4 and 16 weeks. Bacterial genera

present in less than 20% of the samples were excluded from further analysis. All p-values <0.1 were included in the results.

Relative abundance and taxonomic profiles were computed using the Quantitative Insights Into Microbial Ecology (QIIME) tool [8].

Reads containing Illumina Universal Adapters or PhiX were discarded using bbduk version 38.25 (BBTools, https://jgi.doe.gov/data-and-tools/bbtools/) and the remaining reads were demultiplexed using je version 1.2 [9]. Indexes, heterogeneity spacers and primers were trimmed with cutadapt version 1.18 [10] and the paired-end reads were subsequently quality trimmed and merged using bbmerge version 38.25 [11]. The merged contigs were trimmed to 400bp and denoised to retrieve ASV (Amplicon Sequence Variants) with deblur [12] in Qiime2 [13]. ASVs with an abundance <0.005% of the total abundance in the library were discarded. Taxonomic classification of each ASV was done in Qiime2 using a naive bayes classifier [14] trained on a preclustered version (99% sequence similarity) of the Silva database version 132 [15]. Contaminants were determined based on negative correlation with read-depth and was removed (Rhizobiales, Caulobacterales, Chloroplast, Pseudomonadales, Halomonadaceae, Shewanellaceae, Microbacterium, Renibacterium), and a phylogenetic tree was created based on the remaining ASVs. Samples were rarefied (subsampled) to an ASV count of 3875 per sample, and all further analyses were performed on this rarefied dataset. Calculations of alpha diversity (bacterial richness estimate (Observed ASV), Shannon diversity index and Faith's phylogenetic diversity) and beta diversity (unweighted UniFrac) were performed in Qiime2. Differences between paired comparisons was calculated by Mann-Whitney U test.

References:

1. Khanna D, Nagaraja V, Gladue H, Chey W, Pimentel M, Frech T. Measuring Response in the Gastrointestinal Tract in systemic sclerosis. Current opinion in rheumatology. 2013; 25(6):10.1097/1001.bor.0000434668.0000432150.e0000434665.
2. Miller MR, Hankinson J, Brusasco V, Burgos F, Casaburi R, Coates A, et al. Standardisation of spirometry. 2005; 26(2):319-338.
3. Ahlquist DA, Schwartz S, Isaacson J, Ellefson M. A Stool Collection Device: The First Step in Occult Blood Testing. Annals of Internal Medicine. 1988; 108(4):609-612.
4. Valeur J, Røseth AG, Knudsen T, Malmstrøm GH, Fiennes JT, Midtvedt T, et al. Fecal Fermentation in Irritable Bowel Syndrome: Influence of Dietary Restriction of Fermentable Oligosaccharides, Disaccharides, Monosaccharides and Polyols. Digestion. 2016; 94(1):50-56.
5. Planer JD, Peng Y, Kau AL, Blanton LV, Ndao IM, Tarr PI, et al. Development of the gut microbiota and mucosal IgA responses in twins and gnotobiotic mice. Nature. 2016 05/25; 534(7606):263-266.
6. Fadrosh DW, Ma B, Gajer P, Sengamalay N, Ott S, Brotman RM, et al. An improved dual-indexing approach for multiplexed 16S rRNA gene sequencing on the Illumina MiSeq platform. Microbiome. 2014 2014/02/24; 2(1):6.
7. Planer JD, Peng Y, Kau AL, Blanton LV, Ndao IM, Tarr PI, et al. Development of the gut microbiota and mucosal IgA responses in twins and gnotobiotic mice. Nature. 2016 Jun 09; 534(7606):263-266.
8. Moayyedi P, Surette MG, Kim PT, Libertucci J, Wolfe M, Onischi C, et al. Fecal Microbiota Transplantation Induces Remission in Patients With Active Ulcerative Colitis in a Randomized Controlled Trial. Gastroenterology. 2015 Jul; 149(1):102-109.e106.
9. Girardot C, Scholtalbers J, Sauer S, Su S-Y, Furlong EEM. Je, a versatile suite to handle multiplexed NGS libraries with unique molecular identifiers. BMC bioinformatics. 2016; 17(1):419-419.
10. Martin M. Cutadapt removes adapter sequences from high-throughput sequencing reads. EMBnetjournal. 2011 2011-05-02; 17(1):3.
11. Bushnell B, Rood J, Singer E. BBMerge - Accurate paired shotgun read merging via overlap. PloS one. 2017; 12(10):e0185056-e0185056.
12. Amir A, McDonald D, Navas-Molina JA, Kopylova E, Morton JT, Zech Xu Z, et al. Deblur Rapidly Resolves Single-Nucleotide Community Sequence Patterns. mSystems. 2017; 2(2):e00191-00116.
13. Bolyen E, Rideout JR, Dillon MR, Bokulich NA, Abnet C, Al-Ghalith GA, et al. QIIME 2: Reproducible, interactive, scalable, and extensible microbiome data science. PeerJ Preprints. 2018 2018/12/03; 6:e27295v27292.
14. Pedregosa F, Gramfort A, Micael V, Thirion B, Grisel O, Blondel M, et al. Scikit-learn: Machine Learning in Python The Journal of Machine Learning Research. 2011; 12:2825-2830.
15. Quast C, Pruesse E, Gerken J, Peplies J, Yarza P, Yilmaz P, et al. The SILVA ribosomal RNA gene database project: improved data processing and web-based tools. Nucleic Acids Research. 2012; 41(D1):D590-D596.
