# Supplementary material for: Fecal microbiota transplantation in systemic sclerosis: A double-blind, placebo-controlled randomized pilot trial
Source: PLoS One. 2020 May 21;15(5):e0232739. doi: 10.1371/journal.pone.0232739 (PMC7241803; doi:10.1371/journal.pone.0232739)
Supplement: S3 File — (DOCX) [file pone.0232739.s003.docx]

## Aiming to Reduce disease activity in Systemic Sclerosis by cultivated human gut microbiome transplantation; -the controlled ReSScue trial

**Content; (A) Background, (B) study rationale and design, (C) aims, (D) methods (E) collaborators (F) Budget**

1. **Introduction**

SSc is a complex disorder that is highly challenging to manage^1^. Disease risk is multi-factorial*,* with modest contribution by (immune-related) genes and major risk conferred by unknown environmental factors which presumably trigger and drive critical SSc disease processes such as tissue fibrosis, end organ arterial obliteration and induction of anti-nuclear antibody (ANA) subsets^2^. The disease has a **dramatic 25-40 years reduced life expectancy**, mainly caused by progressive and therapy-resistant cardiopulmonary and gastrointestinal (GI) involvement^3^. It is unknown why SSc is resistant to available therapies, but apparent that novel therapeutic strategies are highly desired^1,4^.

ReSScue is a pragmatic therapeutic intervention trial where we will assess a novel treatment strategy for SSc associated GI involvement, test intervention by gut microbiota transplantation (GMT) and investigate molecular effects of GMT on gut, skin and oral microbiomes and on immune cell profiles in these organs. The trial will be performed in collaboration with experienced national and international teams with complementary skills covering all aspects of the project, including core clinical competencies, and excellence in basic sciences.

1. **Background**

**Skin thickening** (scleroderma) is the hallmark of SSc and >90% of SSc patients have skin involvement (**Figure 1**). The **GI tract** is afflicted in 90-100% of the SSc population^5^, with all segments being involved; resulting in a range of severe problems that includes acid regurgitation, malabsorption, chronic diarrhoea and anal incontinence^5^. GI disease is a leading cause of morbidity and a major contributor to poor quality of life in SSc, but there is no treatment other than symptomatic. **Pulmonary hypertension (PH),** diagnosed by invasive right heart catheterization (RHC), develops in 15-20% of SSc patients; with isolated PAH and PH secondary to ILD as the major forms^1^. PAH takes often a rapid and devastating course, ending in right heart failure and death. Approved therapies for PAH target vascular reactivity and remodeling, but are not immune modulating.

**ILD** is currently the leading cause of morbidity and mortality in SSc; and data from the European multicentre database EUSTAR indicate that ILD is present in >50% of the patients^3^. Extent and clinical course of ILD varies; from mild, non-progressive disease, to widespread pro-fibrotic inflammation rapidly progressing to respiratory failure and death. Recent clinical trials applying immune modulating therapies have only shown stabilization of ILD, with no improvement^6,7^.

**Medical therapies** are, as indicated, largely inefficient in SSc, The only major exception is high dose immune-suppression with autologous hematopoietic stem cell transplantation (HSCT), which reduces long-term mortality (and reverses progressive disease in selected cases)^8^. HSCT is, however, only indicated in a small subset of the SSc population, side effects are a concern and costs are high^8^.

| **Gut microbiome** |
| --- |

**The gut microbiome; cross-talk with the immune system and microbiomes in other organs**

Advances in genetic technologies and bioinformatics have made it possible to sequence the trillions of microbes inhabiting the human intestine^9^; and existing results are emerging on interactions between microbiome and immune system, including intestinal Ig, as well as systemic inflammation, metabolism and disease processes in distant organs^10^. The field is still in its infancy, with major unknowns, such as how the microbiome influences intestinal IgA (or IgG) repertoires^11^.

**Potential roles of the gut microbiome in SSc pathogenesis**

In 2015, Volkmann et al from UCLA demonstrated **profound gut microbiome alterations in SSc,** with decreased bacterial diversity and predominance of potentially harmful species^12^. In collaboration with the UCLA, we recently identified similar microbiome alterations in the Nor-SSc cohort^13^. The clinical impact of these findings is not clear, but it is intriguing that a Swedish study found associations between SSc gut microbiome, GI symptoms and intestinal inflammation^14^. There are no data on how the SSc microbiome interacts with intestinal IgA, but we are currently investigating this issue.

**Therapeutic targeting of the gut microbiome; and the rationale for this approach in *SSc***

Therapeutic benefit of GMT is evident in many diseases with gut microbiome dysregulation. The prime example is C. Difficile infection, where 90% of patients are cured of their colitis by GMT with feces from “random” healthy donors^15^. Intriguingly, it appears that donor properties determine clinical efficacy of the “random” feces GMT; indicating distinct specificity of the GMT^16^. No data exist on GMT in SSc, but the approach is rationalized by data from recent studies (**Box 1**).

**Box 1: Scientific rationale for therapeutic gut microbiome transplantation (GMT) in SSc**

- All studies on SSc genetics suggest a dominant role of the environment; and recent analyses indicate that gut microbiome dysregulation confers environmental risk in SSc^1,2^
- The gut microbiome dysregulation in SSc is common to Norwegian and US cases (across ethnicities and diet); indicating primary links between dysregulation and disease pathogenesis^16^.
- Effects of correcting gut microbiome dysregulation appear to include modulation of local and systemic immune networks^17^, both of which are highly relevant for SSc pathogenesis
- Emerging data indicate causal relations between gut microbiome abnormalities, GI pathology and systemic disease activity in human diseases^18,19^; similar causality may also exist in SSc

**3. Hypotheses**

**We hypothesize** that an altered gut microbiome is a key environmental risk factor that drives specific disease process through its effects on local and systemic immune responses; leading to the assumption that normalization of the SSc gut microbiome by standardized GMT intervention should reduce ongoing disease activity.

**4. Material and method**

**Study design**: This is a randomized, double blind clinical trial testing intervention by standardized GMT. The study cohort will include 10 SSc patients with GI symptoms. Evaluation of end points is at 16 weeks. The trial is set up with six study visits (Table 3).

**Target population and eligibility criteria:** We will include SSc patients >18 years who meet the 2013 SSc classification criteria, have objective GI involvement and provide informed consent. As this is an early phase trial, we will exclude patients with severe organ dysfunction (and risk of procedure related complications). As in other GMT studies, we will exclude patients recently exposed to antibiotics. We set no restrictions on concomitant medications.

**Treatment:** 5 patients will receive GMT at baseline and two weeks consisting of duodenal infusion of 30 ml ACHIM by gastroscopy and 5 patients will receive placebo. Small intestinal protocol biopsies will be obtained prior to infusion. Performing GMT with the standardized *in vitro* cultured microbiome ACHIM will allow for systematic donor microbiome tracking; making it feasible to assess effects of the GMT across the study cohort. According to the manufacturer, ACHIM has a genomic diversity and richness comparable to fresh donor faeces; and good clinical efficacy in recurrent C. Difficile colitis^17^. Safety data on ACHIM, available from >400 GMTs indicate few and mild side effects^17^.

**Assessment of primary end point**: As there are no clinical disease activity indices available for SSc that include GI parameters, and no data on defined GI outcomes from any SSc treatment study, we had to define novel outcome measures regarding GI disease for the current study. After careful review of published data we reasoned that it would be most appropriate to apply a patient-reported outcome measure; the validated UCLA GIT version 2.0, and its accompanying descriptions of minimally clinically important differences (MCIDs) - the smallest change in score that patients perceive as beneficial – to define the primary end point for the trial^18,19^. Additionally, we had previously translated this score to Norwegian and had used it for assessment of GI disease in the Nor-SSc cohort (unpublished data).

The UCLA GIT 2.0 assesses GI symptom severity in SSs by 34 items in 7 multi-item scales (reflux, bloating, diarrhea, fecal soilage, constipation, emotional well-being, and social functioning) and a total GIT score^19^. All scales are scored from 0 (better) to 3 (worse) except the diarrhea and constipation (range from 0-2 and 0-2.5, respectively). The score was found to have acceptable feasibility, reliability (test-retest and internal consistency) and validity in large cohorts; and MCID were defined for total GIT score and for the seven individual scales^18^.

For ReSScue we decided to use the 5 individual items related to GI-symptoms and the total score (Table 3). Changes from week 0 to week 16 were defined as positive if the delta change had a p-value<0.05 and was above the MCID defined as “somewhat better” in previous work^18^.

**Table 3: Overview of the UCLA GIT score items applied in ReSScue, with defined baseline threshold values for each item and published data on the magnitude of change defined as minimal clinically important differences (MCID) for each item**

| Item | MCID-somewhat better | MCID-much better |
| --- | --- | --- |
| Reflux | -0.26 (-0.44, -0.07) | -0.35 (-0.69, 0.00) |
| Distention/bloating | -0.04 (-0.44, 0.37) | -0.71 (-1.15, -0.27) |
| Diarrhea | -0.22 (-0.72, 0.28) | -0.41 (-0.74-0.08) |
| Constipation | -0.15 (-0.47, 0.16) | -0.11 (-0.55, 0.32) |
| Fecal soilage | -0.18 (-0.55, -0.20) | -0.36 (-0.82, -0.09) |
| Total GIT score | -0.18 (-0.32, -0.03) | -0.51 (-0.85, -0.16) |

**Preparation, handling and storage of biological material***:* Blood samples will be frozen down and stored in the NOSVAR biobank. Processing of plasma, serum, RNA and DNA will be according to standard procedures. Faecal samples will be collected at home by a specialized (toilet) system, frozen down to -20°C, transported to OUS and stored at -70°C before thawing and DNA isolation by standard procedures. Gut and skin biopsies for immunohistochemistry will be snap-frozen on liquid nitrogen, while skin, gut and oral samples for cell and microbiome analyses will be processed immediately

**Controls and validation populations:** Ten (10) healthy individuals matched to patients by age and gender will be drawn from the population registry and asked to provide faeces, urinary and blood samples at week 0 and 16. Validation will be possible in two cohorts; a Scandinavian SSc cohort from Lund University (LU), possessing 100 faeces samples and an American SSc cohort from UCLA.

**Processing and analyses of DNA from oral skin, and gut samples**: Microbiome composition will be evaluated by deep sequencing of 16S rRNA gene using Illumina MiSeq as described^9^. Beta diversity (Bray-Curtis dissimilarity) will be calculated using Phyloseq R package21 and statistical differences between pairwise comparisons will be calculated using Mann-Whitney U test. Relative abundance and taxonomic profiles will be computed using Quantitative Insights Into Microbial Ecology.

**Analyses of Ig coated intestinal bacteria:** We will apply a method modified from Planer^11^. Briefly, frozen fecal samples are suspended in PBS, vortexed and placed on ice. 5 mg of feces passes through a nylon 70 μm mesh filter, ice cold PBS is added and centrifuged at 10,000*g* for 3 min (4 °C). The resulting supernatant discards and the cell pellet re-suspends in 500µl FACS buffer and aliquot into 5 tubes before addition of 5 µl anti-IgM PE, anti-IgG PE, anti-IgA PE, anti-IgA isotype control or PBS. SytoBC bacterial DNA stain is added and before sort using FACS. For each sample, 50,000 ‘events’ will be recovered from the ‘Input’, ‘Ig+’, and ‘Ig−’ gates.

**Flow cytometry on freshly isolated peripheral blood T-cells and B-cells:** Cells will be sorted by FACS sorting and multi-parameter staining will be performed by standard operating protocols. Relative frequencies and phenotype of IL-17+ cells, IL-13+ cells, T reg cells and B cells and CD14 cells will be assessed in fresh patient and control samples.

**Immunohistochemistry on skin and gut biopsies:** Staining will be performed by Vectastain ABC Elite kits and primary antibodies will be applied and specific labeling detected with a species specific biotinylated secondary antibody and application of horseradish peroxidase-conjugated avidin-biotin followed by development with 3,3′-diaminobenzidine (DAB) solution (Vector).

**Potential blood markers of systemic inflammation level in SSc:** Blood will be frozen down for later analyses of systemic inflammation markers, protein biomarkers and RNA analyses, including plasma LPS, sCD14, sCD25. Chemokines CCL2 and CCL18 will be measured in as described^20^.

**Potential use of skin biopsies:** The skin thickness, immune cell profiles by IHC and in vitro culture Isolation of skin fibroblasts for in vitro use will be conducted on the collected skin biopsies.

**5. Participants, organization and collaborations**

To secure trial feasibility, we have gathered a team of experienced researchers, with international level expertise on their respective fields. They possess complementary skills that cover all the WPs; and they have defined roles and responsibilities (**Table 3**).

**Table 3: Overview of key research personnel in the ReSScue trial**

| **Name** | **Site** | **Position, Field** | **Trial responsibilities** |
| --- | --- | --- | --- |
| Anna Hoffmann-Vold | OUS | Post-Doc, rheumatology | Principal Investigator |
| Øyvind Molberg | OUS | Prof, rheumatology | Head of SSc research |
| Øyvind Midtvedt | OUS | Senior consultant, rheum | Site Investigator, Oslo |
| Knut Lundin | OUS | Prof, gastroenterology | Endoscopy, immune markers |
| Johannes Hov | OUS | Post-Doc, gastroenterology | Microbiome, bioinformatics |
| Pål Aukrust | OUS | Prof, immunology | Immune markers |
| Espen Bekkevold | OUS | Researcher, immunology, | Bacterial IgA, SSc-ILD cell profiling, phenotyping |
| Cathrine Brunborg | OUS | Senior statistician | Statistics |
| May Brit Lund | OUS | Ass Prof, lung medicine | Pulmonary function, BAL |
| Arne Andreassen | OUS | Senior consultant, cardiology | RHC, PAH diagnosis |

**Organization**

Patient recruitment, eligibility screening and retrieval of informed consent will be performed at OUS. At visit 1, 4, 5 and 6 patients will undergo clinical examination, PFTs and sampling of biological material (**Table 4**).

**Table 4: Plan for activities and data acquisition in WP 1**

| **Week** | **-2** | **0** | **2** | **6** | **10** | **16** |
| --- | --- | --- | --- | --- | --- | --- |
| **Visit** | **1** | **2** | **3** | **4** | **5** | **6** |
| **Study inclusion** | **+** |  |  |  |  |  |
| **Intervention** (GMT) |  | **+** | **+** |  |  | **+** |
| Upper GI endoscopy (with biopsies) |  | **+** | **+** |  |  | **+** |
| Validated SSc GI symptom score^1^ |  | **+** | **+** | **+** | **+** | **+** |
| Rodnan skin score |  | **+** |  | **+** |  | **+** |
| Pulmonary function tests |  | **+** |  |  |  | **+** |
| SSc disease status, with 6 CSM |  | **+** |  |  |  | **+** |
| **Samplings of biological material** |  |  |  |  |  |  |
| Fecal microbiota and Calprotectin |  | **+** | **+** | **+** | **+** | **+** |
| Blood and urine for biobank |  | **+** | **+** | **+** | **+** | **+** |
| Oral swab for oral mikrobiota |  | **+** | **+** | **+** | **+** | **+** |
| Skin swabs for skin microbiota |  | **+** | **+** | **+** | **+** | **+** |
| Skin biopsies (for microbiota and cell analyses)^1^ |  | **+** |  |  |  | **+** |

Visits 2 and 3, which will last for two days, the patients will first meet with the trial PI for standardized interviews on recent infections, antibiotics use and SSc disease status before the proceed to examination of skin, sampling of biological material and the primary intervention. After the intervention, the patients will be observed overnight.

**Potential markers of inflammation in blood and feces:** Assessments of blood markers will be conducted in collaboration with the Research Institute of Internal Medicine at OUS. Assessment of Calprotectin in fecal sample will be by ELISA techniques from Calpro.

**Processing and analyses of DNA from oral skin, and gut samples**: These assessments will be conducted in collaboration with Johannes Hov who has excellent competence and available equipment in the field.

**6. Ethical considerations**

The ReSScue trial was approved by the Regional Committee for Medical Research Ethics in South-East Norway (2016/1529). It will be conducted in accordance with the “Declaration of Helsinki” and “Guidelines of good Clinical Practice” and the study will be reported to ClinicalTrials.gov.

**7. References**

1. Wigley FM, Boin F. Chapter 84 - Clinical Features and Treatment of Scleroderma A2 - Firestein, Gary S. In: Budd RC, Gabriel SE, McInnes IB, O'Dell JR, eds. Kelley and Firestein's Textbook of Rheumatology (Tenth Edition): Elsevier; 2017: 1424-60.e5.

2. Bossini-Castillo L, Lopez-Isac E, Martin J. Immunogenetics of systemic sclerosis: Defining heritability, functional variants and shared-autoimmunity pathways. *Journal of autoimmunity* 2015; **64**: 53-65.

3. Tyndall AJ, Bannert B, Vonk M, et al. Causes and risk factors for death in systemic sclerosis: a study from the EULAR Scleroderma Trials and Research (EUSTAR) database. *Annals of the rheumatic diseases* 2010; **69**(10): 1809-15.

4. Pattanaik D, Brown M, Postlethwaite BC, Postlethwaite AE. Pathogenesis of Systemic Sclerosis. *Front Immunol* 2015; **6**: 272.

5. Kumar S, Singh J, Rattan S, DiMarino AJ, Cohen S, Jimenez SA. Review article: pathogenesis and clinical manifestations of gastrointestinal involvement in systemic sclerosis. *Alimentary pharmacology & therapeutics* 2017; **45**(7): 883-98.

6. Tashkin DP, Roth MD, Clements PJ, et al. Mycophenolate mofetil versus oral cyclophosphamide in scleroderma-related interstitial lung disease (SLS II): a randomised controlled, double-blind, parallel group trial. *The Lancet Respiratory medicine* 2016; **4**(9): 708-19.

7. Khanna D, Denton CP, Jahreis A, et al. Safety and efficacy of subcutaneous tocilizumab in adults with systemic sclerosis (faSScinate): a phase 2, randomised, controlled trial. *Lancet (London, England)* 2016; **387**(10038): 2630-40.

8. van Laar JM, Farge D, Sont JK, et al. Autologous hematopoietic stem cell transplantation vs intravenous pulse cyclophosphamide in diffuse cutaneous systemic sclerosis: a randomized clinical trial. *Jama* 2014; **311**(24): 2490-8.

9. Kummen M, Holm K, Anmarkrud JA, et al. The gut microbial profile in patients with primary sclerosing cholangitis is distinct from patients with ulcerative colitis without biliary disease and healthy controls. *Gut* 2017; **66**(4): 611-9.

10. Schroeder BO, Backhed F. Signals from the gut microbiota to distant organs in physiology and disease. *Nature medicine* 2016; **22**(10): 1079-89.

11. Planer JD, Peng Y, Kau AL, et al. Development of the gut microbiota and mucosal IgA responses in twins and gnotobiotic mice. *Nature* 2016; **534**(7606): 263-6.

12. Volkmann ER, Chang YL, Barroso N, et al. Association of Systemic Sclerosis With a Unique Colonic Microbial Consortium. *Arthritis & rheumatology (Hoboken, NJ)* 2016; **68**(6): 1483-92.

13. Volkmann ER, Hoffmann-Vold AM, Chang YL, et al. Systemic sclerosis is associated with specific alterations in gastrointestinal microbiota in two independent cohorts. *BMJ open gastroenterology* 2017; **in press**.

14. Andreasson K, Alrawi Z, Persson A, Jonsson G, Marsal J. Intestinal dysbiosis is common in systemic sclerosis and associated with gastrointestinal and extraintestinal features of disease. *Arthritis Res Ther* 2016; **18**(1): 278.

15. van Nood E, Vrieze A, Nieuwdorp M, et al. Duodenal infusion of donor feces for recurrent Clostridium difficile. *The New England journal of medicine* 2013; **368**(5): 407-15.

16. Moayyedi P, Surette MG, Kim PT, et al. Fecal Microbiota Transplantation Induces Remission in Patients With Active Ulcerative Colitis in a Randomized Controlled Trial. *Gastroenterology* 2015; **149**(1): 102-9.e6.

17. Norin E. Experience with cultivated microbiota transplant: ongoing treatment of Clostridium difficile patients in Sweden. *Microbial ecology in health and disease* 2015; **26**: 27638.

18. Khanna D, Furst DE, Maranian P, et al. Minimally important differences of the UCLA Scleroderma Clinical Trial Consortium Gastrointestinal Tract Instrument. *The Journal of rheumatology* 2011; **38**(9): 1920-4.

19. Khanna D, Hays RD, Maranian P, et al. Reliability and validity of the University of California, Los Angeles Scleroderma Clinical Trial Consortium Gastrointestinal Tract Instrument. *Arthritis and rheumatism* 2009; **61**(9): 1257-63.

20. Hoffmann-Vold AM, Tennoe AH, Garen T, et al. High Level of Chemokine CCL18 Is Associated With Pulmonary Function Deterioration, Lung Fibrosis Progression, and Reduced Survival in Systemic Sclerosis. *Chest* 2016; **150**(2): 299-306.
