# Supplementary material for: Fecal microbiota transplantation in systemic sclerosis: A double-blind, placebo-controlled randomized pilot trial
Source: PLoS One. 2020 May 21;15(5):e0232739. doi: 10.1371/journal.pone.0232739 (PMC7241803; doi:10.1371/journal.pone.0232739)
Supplement: S5 File — (PDF) [file pone.0232739.s005.pdf]

ACHIM

ACHIM is produced by ACHIM AB biotherapeutics (556939-7788), Sweden. Anaerobically cultivated Human Intestinal Microbiome (ACHIM) is a standardized human fecal microbiota composition. The microbiota originates from feces donated back in 1995, from a single feces donor. The donor was healthy and fulfilled the same criteria's used in a feces bank.

The microbiota culture in ACHIM has been cultivated strictly anaerobically. Weekly inoculations since 1995 has been performed. ACHIM is thoroughly tested and does not contain viruses or human cells. The LOT number of the ACHIM used in this study was 20171004.
