# Supplementary figures and images for: Fecal microbiota transplantation in systemic sclerosis: A double-blind, placebo-controlled randomized pilot trial
Source: PLoS One. 2020 May 21;15(5):e0232739. doi: 10.1371/journal.pone.0232739 (PMC7241803; doi:10.1371/journal.pone.0232739)

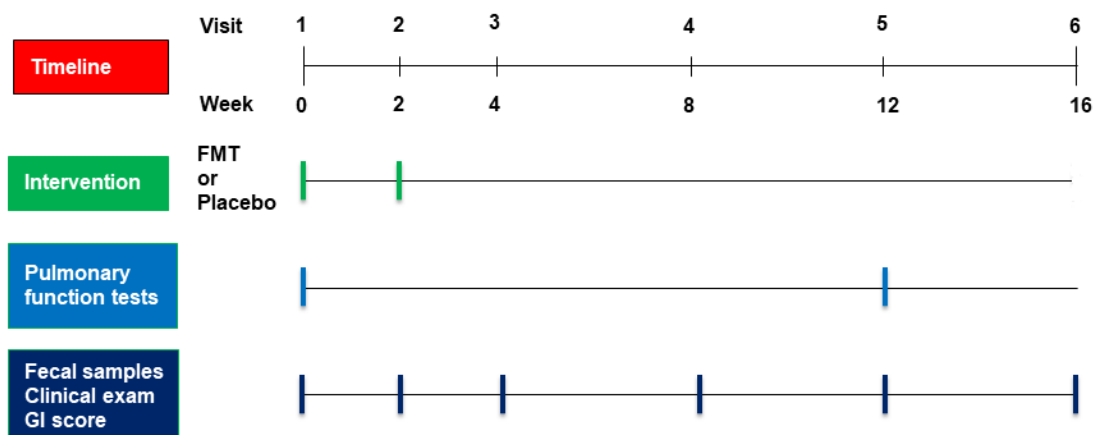

Supplement: S1 Fig — Six study visits over a period of 16 weeks. Intervention with ACHIM with two weeks apart (week 0 and 2). Pulmonary function tests performed at week 0 and 12. Fecal samples each week, and collected at all study visits. Clinical exam and UCLA GIT score performed at all study visits (week 0, 2, 4, 8, 12 and 16). (PDF) [file pone.0232739.s007.pdf]

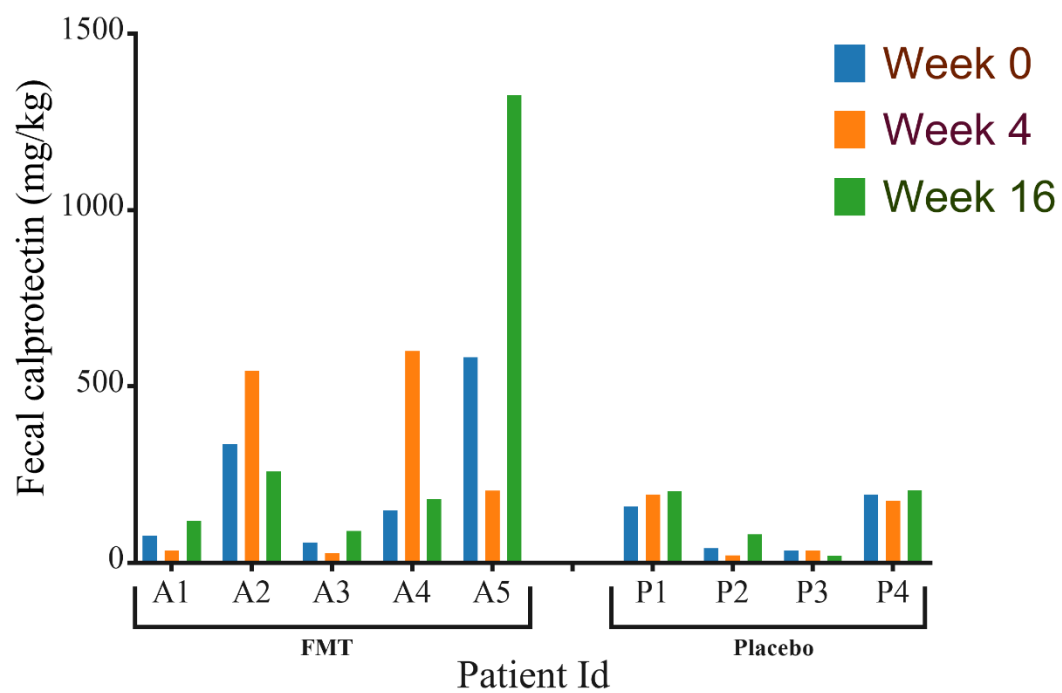

Supplement: S2 Fig — Individual level of fecal calprotectin (mg/kg) at week 0, week 4 and week 16. A = active treatment group patient. P = placebo group patient. (PDF) [file pone.0232739.s008.pdf]

Beta-diversity PCoA  
Unweighted Unifrac

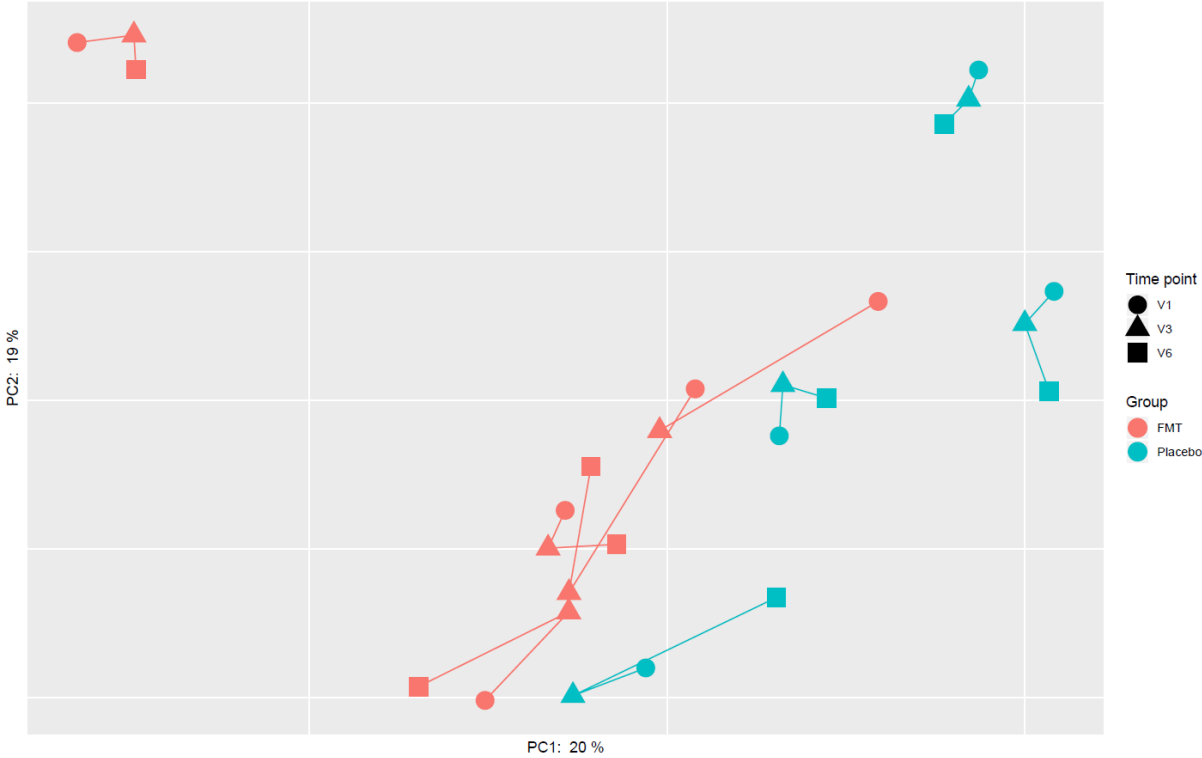

Supplement: S3 Fig — Individual beta diversity at week 0 (V1), week 4 (V3) and week 16 (V6). (PDF) [file pone.0232739.s009.pdf]
